# Evaluating lipid-driven insulin resistance via TyG index in breast cancer patients: Toward effective secondary prevention

**DOI:** 10.3205/000347

**Published:** 2025-10-01

**Authors:** Bandana Kumari, Rijhul Lahariya

**Affiliations:** 1 Department of Biochemistry, All India Institute of Medical Sciences, Patna, Bihar, India; 2 All India Institute of Medical Sciences, Patna, Bihar, India

**Keywords:** lipid profile, triglycerides, insulin resistance, breast neoplasm, logistic regression

## Abstract

**Objective:**

Breast cancer is the most commonly diagnosed malignancy worldwide. Insulin resistance (IR) plays a key role in its progression by activating oncogenic signaling pathways. The triglyceride-glucose (TyG) index is a validated, cost-effective surrogate marker for IR. This study aims to evaluate the prevalence of IR in female breast cancer patients using the TyG index and to identify lipid parameters associated with increased IR, thereby supporting strategies for secondary prevention.

**Methods:**

A cross-sectional study was conducted among non-diabetic, histopathologically confirmed female breast cancer patients. Demographic data, lipid profiles, and fasting glucose levels were collected. Participants were stratified into high-risk (TyG≥8.87) and low-risk (TyG<8.87) groups based on their TyG index. Logistic regression analysis was performed to identify significant predictors of elevated TyG index.

**Results:**

Among 122 patients, 44.3% demonstrated elevated insulin resistance. Triglycerides (TG), total cholesterol (TC), very low-density lipoprotein cholesterol (VLDL-C), and the TC/high-density lipoprotein-cholesterol (HDL-C) ratio were significantly higher in the high-risk group. Logistic regression identified TC, TC/HDL-C ratio, and low-density lipoprotein cholesterol (LDL-C) as significant predictors of elevated IR (p<0.05). The model is represented as: Logit(P)=–13.941+0.145X_1_+1.558X_2_–0.178X_3_, where X_1_, X_2_, and X_3_ correspond to TC, TC/HDL-C ratio, and LDL-C, respectively. The predictive model achieved 90.2% accuracy with an area under the receiver operating characteristic (ROC) curve (AUROC) of 0.927.

**Conclusion:**

Monitoring lipid parameters and managing insulin resistance are crucial for enhancing breast cancer prognosis and potentially reducing progression.

## Introduction

According to the 2020 GLOBOCAN Global Cancer Report, breast cancer has surpassed lung cancer as the most frequently diagnosed cancer in females, with an estimated 2.3 million new cases reported [1]. The situation is alarming as every 4 minutes a woman is diagnosed with breast cancer, and one woman dies from the disease every 13 minutes, largely due to late-stage diagnosis or improper prevention [2], [3]. Projections suggest that by 2030, breast cancer will become the leading cause of death among Indian women, highlighting the urgent need for effective prevention and early detection strategies [3]. Triple-negative breast cancer (TNBC) is associated with the poorest prognosis among all breast cancers [4]. 

It is well established that poor metabolic health increases the risk of developing breast cancer. However, recent research has shown that body mass index (BMI) alone is not a reliable indicator of metabolic health [5]. For instance, individuals with obesity (BMI>30) can still be metabolically healthy, while those who are lean (BMI<25) may experience metabolic dysfunction [5]. This highlights the importance of considering lifestyle factors – such as diet, physical activity, and stress management – in the prevention and management of metabolic health to reduce the risk of breast cancer [5]. 

Insulin resistance (IR) elevates insulin levels, activating pathways that promote aggressive breast cancer biology. Research on insulin analogues and anti-insulin receptor antibodies highlights the role of insulin signaling in cancer progression. IR exacerbates tumor growth by sustaining hyperinsulinemia, enhancing tumor cell proliferation and survival. Targeting IR through lifestyle changes or pharmacological interventions offers a promising strategy to improve breast cancer outcomes [6]. Additionally, IR contributes to metabolic syndrome, complicating breast cancer management. The PI3K-AKT-mTOR pathway, involved in insulin signaling, mediates resistance to endocrine therapy (ET), with high insulin receptor expression linked to poor prognosis [6].

Not all breast cancer patients exhibit insulin resistance (IR). A study by Alan et al. involving 55 patients found that 25 did not have IR and showed a better response to treatment [7]. Breast epithelial cells are surrounded by adipose tissue, and the crosstalk between these compartments is increasingly recognized as a key factor in the initiation and progression of breast cancer, alongside IR [8]. Lipid dysregulation, a common consequence of IR, leads to free fatty acid accumulation, heightening oxidative stress by increasing reactive oxygen species (ROS) production [9]. This oxidative stress damages DNA, proteins, and lipids, while the hypoxic tumor environment further exacerbates lipid accumulation in breast tissue [10]. This creates a vicious cycle that not only amplifies oxidative stress but also disrupts insulin signaling, promoting tumor growth and metastasis [10]. However, not all lipid parameter derangements predispose individuals to breast cancer. A meta-analysis of 26 prospective studies revealed conflicting data on the relationship between lipids and breast cancer [11].

The Homeostatic Model Assessment of Insulin Resistance (HOMA-IR), based on fasting glucose and insulin levels, is commonly used to evaluate IR. However, the triglyceride-glucose (TyG) index, calculated from fasting triglycerides and glucose, has emerged as a more cost-effective and reliable alternative [12], [13], [14]. This study aimed to assess the prevalence of insulin resistance in female breast cancer patients and identify the lipid parameters most significantly influencing IR to prevent further complications.

## Materials and methods

This retrospective observational study was conducted at a tertiary care center in India. Newly diagnosed, histopathologically confirmed non-diabetic female breast cancer patients aged above 18 years, who provided informed consent and had complete clinical data, were included. Out of 269 patients initially enrolled, only 122 met the inclusion criteria. Patients with diabetes mellitus, those who declined consent, or those with incomplete data were excluded.

Data were collected using a pre-defined proforma covering age, BMI, smoking history, hypertension history, and family history of breast cancer. Laboratory parameters, including lipid profiles and fasting glucose, were retrieved from the hospital’s information system. Based on Panigoro et al., who identified a TyG index >8.87 as a risk threshold for breast cancer [15], participants were categorized into high- and low-risk groups accordingly.

Group I: Breast cancer patients with TyG index <8.87

Group II: Breast cancer patients with TyG index ≥8.87 

The TyG index was calculated as the natural logarithm (Ln) of the product of plasma glucose and triglyceride (TG) using the formula [16]: 

**Ln (TG[mg/dL]×glucose [mg/dL]/2)**

Difference relating to the levels of various lipid parameters and the demographic details were assessed between these two groups.

### Statistical analysis plan

The data was analysed using Jamovi version 2.4.11. Normality of continuous variables was assessed with Q-Q plots and the Shapiro-Wilk test. Continuous variables were reported as mean (±SD)/median (IQR), and categorical variables as frequency and percentage. Subjects were grouped by TyG index (cutoff: 8.87). Differences between groups were analysed using independent t-test or Mann-Whitney U test for continuous variables and Chi-square test for categorical variables. A p-value ≤0.05 was considered significant. Multicollinearity among lipid parameters was assessed using the Variance Inflation Factor (VIF).

Univariate analysis was conducted to identify potential predictors, and significant variables were selected using the backward elimination method for the model. The model was developed using Python version 3.10.12, and its predictive performance was assessed to ensure goodness of fit. Odds ratios with 95% confidence intervals and standard errors were calculated for each variable. The model’s predictive value was evaluated using the receiver operating characteristic (ROC) curve, and a logistic regression formula was derived to estimate the likelihood of insulin resistance in breast cancer patients.

Various Python libraries were utilized, including NumPy for efficient numerical operations, Pandas for handling tabular data, matplotlib.pyplot for creating visualizations such as graphs, sklearn.linear_model for logistic regression modeling, sklearn.metrics for model evaluation, and statsmodels.api for advanced statistical modeling and interpretation of logistic regression results.

## Results

A total of 122 breast cancer patients were recruited, with a mean age of 50.7±10.9 years (range: 26–85 years). Table 1 (Tab. 1) presents the demographic and clinical characteristics of the subjects, categorized by TyG index using a cut-off value of 8.87. There were 54 (44.3%) breast cancer patients with high IR as assessed by TyG index in our cohort. No significant differences were observed between the two groups in terms of age, BMI, history of hypertension, smoking history, low-density lipoprotein cholesterol (LDL-C) levels, high-density lipoprotein cholesterol (HDL-C) levels, LDL-C/HDL-C ratio, or family history. However, the mean or median levels of triglycerides (TG), total cholesterol (TC), very low-density lipoprotein cholesterol (VLDL-C), total cholesterol/HDL-C ratio, VLDL-C/HDL-C ratio, and triglyceride/HDL-C ratio were significantly higher in the elevated TyG index group (Table 1 (Tab. 1)).

Table 2 (Tab. 2) presents the results of the univariate analysis used to identify potential parameters associated with low and elevated TyG index groups for the binary logistic regression model. Among the parameters analysed, TC, VLDL-C, TC/HDL-C ratio, VLDL-C/HDL-C ratio, and TG/HDL-C ratio showed statistical significance (P≤0.05*). Conversely, age, BMI, LDL-C, HDL-C, LDL-C/HDL-C ratio, history of hypertension, family history, and smoking history did not exhibit statistically significant differences (P>0.05).

The multivariate logistic regression analysis found TC, TC/HDL-C ratio, and LDL-C as factors influencing the low and high TyG index groups in female breast cancer patients. We found no multicollinearity among these three parameters as assessed by VIF (less than 10). Thus, our results indicate that only these three are significantly associated with an IR level greater than 8.87, which is a known risk factor for the development of female breast cancer.

As shown in Table 3 (Tab. 3), the predictive model is expressed as: Logit (P)=–13.941+0.145X_1_+1.558X_2_–0.178X_3_, where the covariates X_1_, X_2_ and X_3_ represent TC, TC/HDL-C ratio, and LDL-C, respectively. So, in this logistic regression formula, on putting the values of these three parameters, we will get the logit (P) value, which will be the probability percentage of having high TyG index, indicating higher IR. 

**Figure 1:**
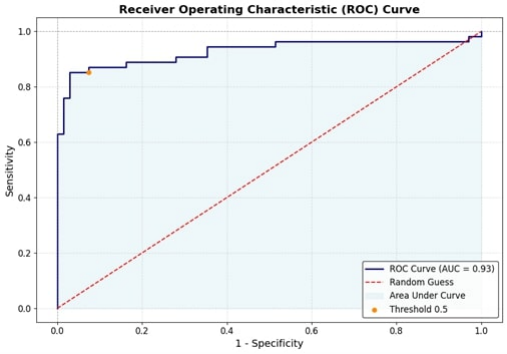
ROC curve of the predictive model with the area under the curve 0.927

The logistic regression model demonstrated excellent predictive performance, with an area under the curve (AUC) of 0.927 (95% CI: 0.864–0.978), indicating strong discriminative ability between high and low TyG index groups. The model showed high sensitivity (85.19%) and specificity (94.12%), with a positive predictive value of 92.00% and a negative predictive value of 88.89%, resulting in an overall accuracy of 90.16%. Additionally, McFadden’s R² value of 0.39 suggests a good model fit. These findings indicate that the model reliably identifies insulin resistance using key lipid parameters. Figure 1 (Fig. 1) shows the ROC curve for the logistic regression model.

## Discussion

Insulin resistance (IR) is increasingly recognized as a contributor to breast cancer progression, with the triglyceride-glucose (TyG) index serving as a reliable marker [1]. Among 122 female breast cancer patients, 44.3% exhibited high IR. The high IR group had elevated triglycerides, total cholesterol (TC), VLDL-C, and TC/HDL-C ratio. Univariate analysis identified TC, VLDL-C, and TC/HDL-C ratio as significant predictors. Multivariate regression confirmed TC, TC/HDL-C ratio, and LDL-C as independent predictors. The model demonstrated strong predictive performance (AUROC=0.927), emphasizing the clinical utility of lipid markers in IR assessment.

Chronic hyperinsulinemia promotes oncogenesis by activating cellular signaling pathways that drive growth factor-dependent cell proliferation. Insulin increases IGF-1 bioactivity by enhancing its production and suppressing IGFBP-1 and IGFBP-2 [17]. It also reduces SHBG levels, elevating free sex hormones like oestradiol and testosterone, which promote breast epithelial proliferation and inhibit apoptosis [17]. Additionally, insulin resistance (IR) induces oxidative stress via excess ROS, contributing to DNA damage and carcinogenesis [17]. Given the abundance of adipose tissue in the breast, IR, adipose-derived oestrogen, inflammatory mediators, and adipokines further amplify cancer risk, particularly in dense breast tissue, by driving oxidative stress and oncogenic signaling [8].

Dysregulated lipid metabolism has been implicated in breast cancer development, although recent meta-analyses have yielded conflicting results regarding the relationship between lipid profile parameters and breast cancer risk [14], [18]. One analysis reported a significant negative association between HDL-C levels and breast cancer risk, while no such correlation was found for TC, TG, or LDL-C [11]. Contrarily, research by Høyer et al. established a significant link between HDL-C levels and breast cancer risk, in contrast to studies by Moorman et al., Furberg et al., and Kucharska-Newton et al., which found no such association [19], [20], [21], [22]. Further investigations, including those by Vatten et al., Gaard et al., and Manjer et al., reported no significant connection between lipid measures and breast cancer prognosis [23], [24], [25]. Additionally, studies by Iso et al. and Fagherazzi et al. identified a significant correlation between TC levels and the increased risk of breast cancer complications, while other studies by Steenland et al. and Eliassen et al. found no such link [26], [27], [28], [29]. Similarly, Melvin et al. highlighted TG levels as significantly associated with higher breast cancer risk and poor prognosis, whereas Ulmer et al. found no such significant relationship [30], [31].

Our study identifies total cholesterol (TC), the TC/HDL-C ratio, and LDL-C as significant factors influencing insulin resistance (IR), as assessed by the TyG index, in female breast cancer patients. We observed that alterations in these lipid parameters are strongly associated with elevated IR, particularly when the TyG index exceeds 8.87, which is linked to a higher risk of complications and poorer prognosis, as highlighted by Panigoro et al. [15]. However, the study has limitations, including a relatively small sample size and its single-center design, which may impact the generalizability of the findings. Further research with larger, multicentre cohorts is needed to better understand the pathogenesis of complications in breast cancer patients. Early prevention and management of insulin resistance through lifestyle changes, including dietary modifications, physical activity, and pharmacological interventions, could play a crucial role in improving patient outcomes and preventing the progression of complications, ultimately leading to better prognosis in breast cancer.

## Conclusion

Our study underscores the pivotal role of lipid abnormalities – specifically total cholesterol (TC), the TC/HDL-C ratio, and LDL-C – in modulating insulin resistance (IR) among women with breast cancer. We observed that these lipid imbalances are significantly linked to elevated IR, as indicated by a TyG index greater than 8.87, a well-established marker of heightened breast cancer risk. Disruptions in lipid metabolism contribute to the pathophysiology of breast cancer, potentially accelerating disease progression. These findings suggest that addressing lipid abnormalities could offer a powerful tool for early detection and preventive strategies. Regular monitoring of lipid profiles, especially for women over 30 or those perimenopausal, is critical to maintaining healthy TC, TC/HDL-C, and LDL-C levels, thereby reducing the risk of breast cancer. Moving forward, larger, multicentre studies are needed to validate these findings and explore the mechanisms by which lipid imbalances impact breast cancer outcomes. In doing so, we can improve prevention efforts and ultimately enhance patient prognosis.

## Highlights

- Our study highlights the significant association between lipid abnormalities – particularly total cholesterol (TC), the TC/HDL-C ratio, and LDL-C – and elevated insulin resistance (IR) in women with breast cancer.
- Elevated IR, as indicated by a TyG index greater than 8.87, was strongly linked to an increased risk of breast cancer progression, emphasizing the role of lipid dysregulation in the disease’s pathophysiology.
- Regular monitoring of lipid profiles, particularly TC, TC/HDL-C, and LDL-C, is essential for early detection and prevention strategies, especially for women over 30 or those perimenopausal.

## Data

Data for this article are available from Dryad [32] (https://doi.org/10.5061/dryad.kd51c5bjn).

## Notes

### Acknowledgement

We acknowledge the residents of our department for providing language help and proof reading the article.

### Ethical statement

This study was approved by the Institutional Ethics Committee of All India Institute of Medical Sciences, Patna (Ref. No. AIIMS/Pat/IEC/2021/804). The requirement for informed consent was waived by the committee. All data were anonymized to maintain patient confidentiality, and the study was conducted in accordance with the ethical standards of the Declaration of Helsinki.

### Authors’ contributions

Bandana Kumari contributed to the study conception, methodology and draft preparation. Bandana Kumari and Rijhul Lahariya contributed in data collection. Data analysis was performed by Rijhul Lahariya. The first draft of the manuscript was written by Bandana Kumari and Rijhul Lahariya. Both authors read and approved the final manuscript. **Rijhul Lahariya and Bandana Kumari contributed equally to this work.**

### Authors’ ORCIDs

- Bandana Kumari: 0000-0001-5395-413X
- Rijhul Lahariya: 0009-0003-5769-4509

### Competing interests

The authors declare that they have no competing interests.

## Figures and Tables

**Table 1:**
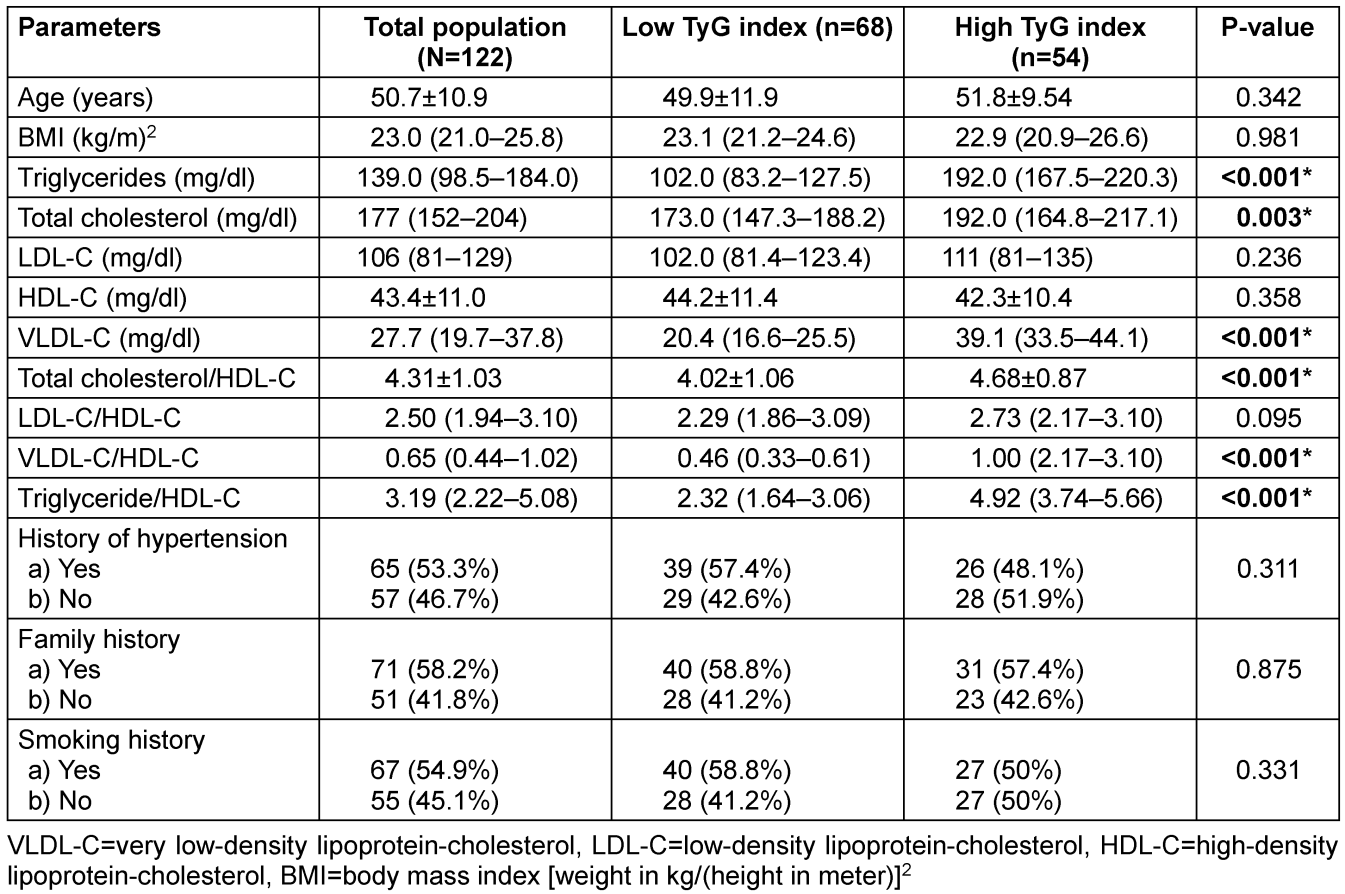
Demographic and clinical characteristics of subjects categorized by TyG index

**Table 2:**
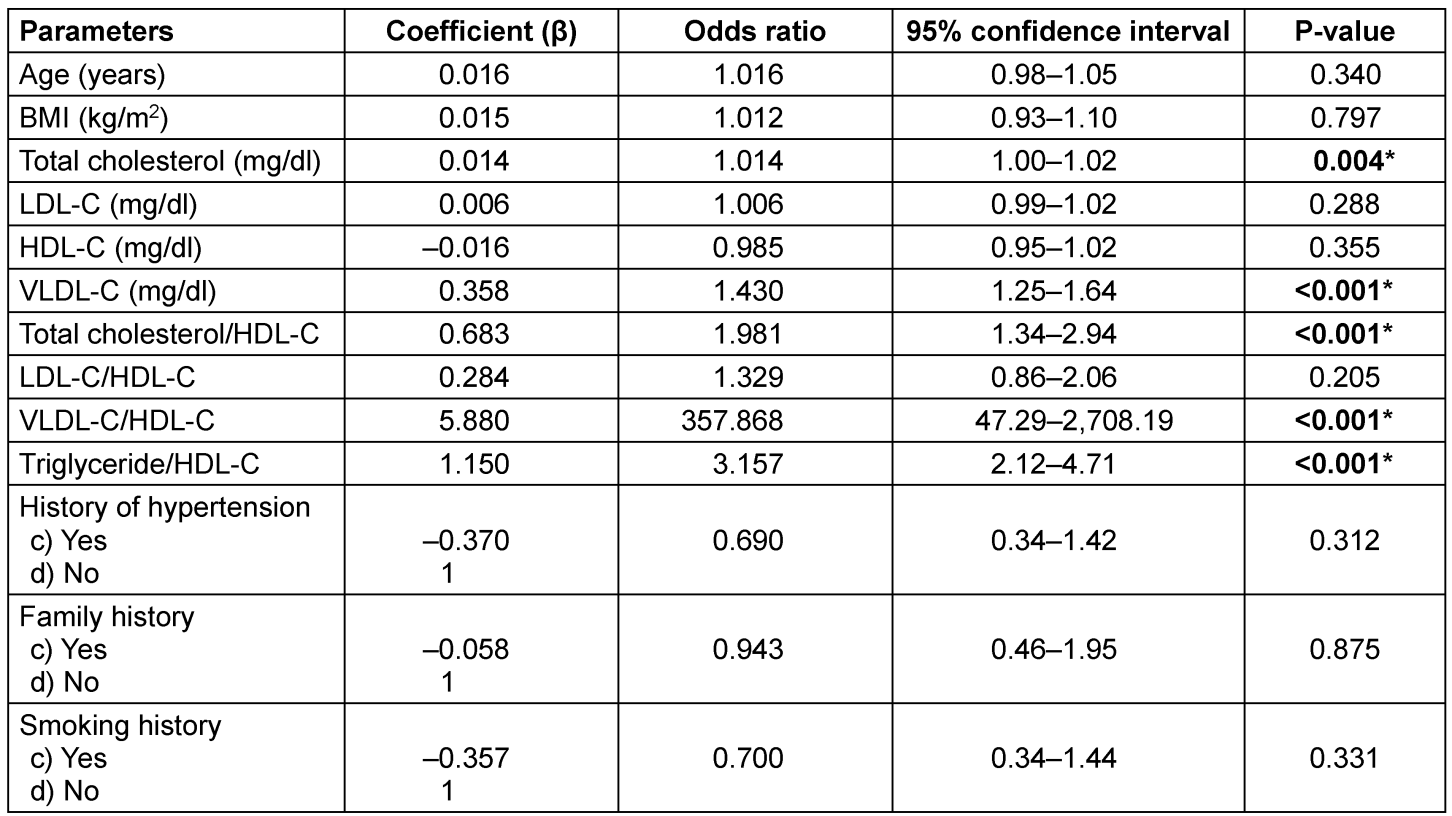
Univariate logistic regression analysis of parameters for predicting insulin resistance by TyG index

**Table 3:**
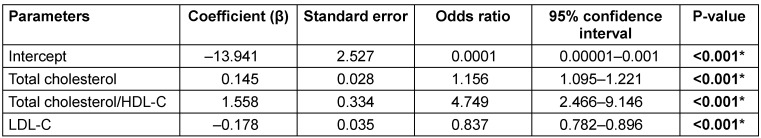
Multivariate logistic regression analysis of parameters for predicting insulin resistance
